# Extranodal marginal zone B‐cell lymphoma of mucosa‐associated lymphoid tissue in the oromaxillofacial head and neck region: A retrospective analysis of 105 patients

**DOI:** 10.1002/cam4.2681

**Published:** 2019-11-15

**Authors:** Tian Zhang, Yunteng Wu, Houyu Ju, Jian Meng, Wei Guo, Guoxin Ren

**Affiliations:** ^1^ Department of oromaxillofacial head and neck region Oncology Shanghai Ninth People's Hospital Shanghai Jiao Tong University School of Medicine Shanghai China; ^2^ Department of Stomatology Central Hospital of Xuzhou Xuzhou Clinical College of Xuzhou Medical University Xuzhou Jiangsu China

**Keywords:** Ann Arbor staging, MALT lymphoma, MALT‐IPI, oromaxillofacial head and neck region

## Abstract

**Background:**

Extranodal marginal zone B‐cell lymphoma of mucosa‐associated lymphoid tissue (MALT lymphoma) in the oromaxillofacial head and neck region is rare, with limited data available. This retrospective study explored the clinical features, stage, treatment, and prognosis of this disease.

**Methods:**

Overall, 105 patients with MALT lymphomas in the oromaxillofacial head and neck region were included in this retrospective analysis. SPSS 22.0 software package was used for data analysis and a two‐tailed *P* value of ≤.05 was considered statistically significant. Primary endpoints of the study were the complete response (CR) rate, overall survival (OS), and progression‐free survival (PFS).

**Results:**

About 52% of the patients had long‐term xerostomia, autoimmune diseases, or chronic parotitis and 81% had diseases involving the large salivary glands. Ann Arbor staging of the patients was as follows: stages I/II, 73 patients and stages III/IV, 32 patients. In the 97 patients followed up, CR rate after initial treatment was 80%. Tumor progression was observed in 12 patients and 14 patients died. There was a significant difference between the rate of CR in localized (87%) and disseminated (67%) lymphoma patients (*P* = .02). The 5‐ and 10‐year PFS of the localized lymphoma patients were both 91%, whereas those of the disseminated lymphoma patients were 83% and 65%, respectively (*P* = .03). The 5‐year PFS rates of the chemotherapy and non‐chemotherapy groups in the disseminated lymphoma patients were 85% and 73% (*P* = .04). Meanwhile, the 5‐year PFS rates of the rituximab and non‐rituximab groups in the disseminated lymphoma patients were 100% and 70% (*P* = .03). In multivariate analysis, MALT Lymphoma International Prognostic Index (MALT‐IPI) was an independent prognostic factor affecting OS, whereas Ann Arbor staging affected PFS.

**Conclusions:**

This study suggests that the outcome after initial treatment of MALT lymphomas in the oromaxillofacial head and neck region is satisfactory and that this disease progresses slowly. The CR rate and PFS of localized lymphoma patients are better than those of disseminated lymphoma patients. Systemic treatment (chemotherapy or rituximab) may improve PFS in disseminated disease patients. MALT‐IPI and Ann Arbor staging are independent prognostic factors.

## Graphical abstract

Systemic therapy may improve survival in disseminated disease patients of MALT lymphomas in the oromaxillofacial head and neck region; MALT‐IPI and Ann Arbor staging are independent prognostic factors.

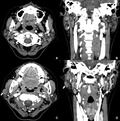

## 1 INTRODUCTION

Extranodal marginal zone B‐cell lymphoma of mucosa‐associated lymphoid tissue (MALT lymphoma) was first described by Isaacson and Wright in 1983^1^ and recognized as a discrete entity in 1994 in the Revised European‐American Lymphoma classification.^2^ MALT lymphomas belong to marginal zone B‐cell lymphomas outside the lymph nodes (accounting for approximately 70%). They are the third most common subtype (7%–8%) of non‐Hodgkin's lymphoma (NHL) after diffuse large B‐cell and follicular lymphomas,^3^ which are also the most common indolent lymphomas in the Chinese.^4^ In general, MALT lymphomas exhibit an indolent behavior and tend to remain localized for a long time, with a high response rate to therapeutic modalities, late relapse, and long overall survival (OS).^5^, ^6^, ^7^

The oromaxillofacial head and neck region is the second most common site of malignant lymphomas. Malignant lymphomas in this region account for approximately 5% of all malignant tumors in the head and neck,^8^, ^9^ among which MALT lymphomas are the most common extranodal B‐cell lymphomas. The most common site of MALT lymphomas is the salivary glands, whereas other sites such as the ocular adnexa and thyroid can also be involved.^10^ The causes of the disease are mostly related to persistent immune stimulation caused by chronic infection or inflammation, such as Hashimoto's thyroiditis and Sjögren's syndrome (SS).^11^

Findings have shown that the clinical features, diagnosis, identification, and biological behavior of MALT lymphomas in the oromaxillofacial head and neck region differ somewhat from those of MALT lymphomas in other sites. Published reports^12^, ^13^ about MALT lymphomas have mainly focused on gastric MALT lymphomas. Primary MALT lymphomas in the oromaxillofacial head and neck region are rare; therefore, the relevant data available in the literature are scarce and confined to small series; the characteristics and clinical outcome of this unique disease thus remain controversial. Against this background, the purpose of this study was to provide more comprehensive evidence focused on primary MALT lymphomas in the oromaxillofacial head and neck region for clinical practice.

## 2 MATERIALS AND METHODS

### 2.1 Participants and sample selection

Approval for this study was obtained from department of oromaxillofacial head and neck region oncology in Shanghai Ninth People's Hospital, all included patients, and their relatives. This retrospective study included 105 patients diagnosed with primary MALT lymphoma in the oromaxillofacial head and neck region between July 2005 and December 2017 at our institution. A total of 97 patients were followed up at the deadline.

Eligible patients were aged ≥18 years or older and had histologically confirmed by two professional oral pathologists, complete medical history and examination data, and an Eastern Cooperative Oncology Group (ECOG) performance status score of ≤2. Key exclusion criteria were previous lymphatic neoplasm or other malignancies and pregnant or lactating women. The data extracted from medical records and retrospective review included demographic information, clinical symptoms, laboratory data, histological characteristics, imaging, performance stage, treatment parameters, recurrence, and survival.

### 2.2 Assessments

The diagnosis of MALT lymphomas was confirmed in all patients according to the criteria proposed initially in the REAL classification^2^ and the 2008 version of the WHO classification of tumors of hematopoietic and lymphoid tissues.^14^ Performance status was evaluated according to the ECOG score. The disease stage was evaluated using Ann Arbor staging,^15^ which was based on findings from computed tomography, magnetic resonance imaging, ultrasound, or positron emission tomography‐computed tomography of the head and neck, thorax, abdomen, and pelvis, bone marrow aspiration, and biopsy. All autoimmune diseases were self‐reported in medical records and nearly all were requested to provide personal history of physician‐diagnosed autoimmune conditions.

MALT Lymphoma International Prognostic Index (MALT‐IPI)^16^ constructed using three parameters (Ann Arbor stage III or IV, age ≥70 years, and elevated LDH levels) identified three groups: low, intermediate, and high risk (corresponding to the presence of none, one, or two or more of these factors, respectively).

Treatment response was assessed according to the standardized criteria reported in the international workshop for NHL.^17^, ^18^ Complete response (CR) was defined as complete disappearance of all detectable clinical evidence of disease and disease‐related symptoms if present before therapy and the CR rate was defined as the percentage of cases of CR in the total number of cases treated.

OS was defined as the time from diagnosis (first biopsy) to death or last follow‐up. Progression‐free survival (PFS) was defined as the time from onset of treatment to the date of the first progression or last follow‐up.

Chi‐squared and Fisher's exact tests were used in univariate analysis to determine the significance of differences between constituent ratios. Actuarial survival curves were calculated using the Kaplan‐Meier method, and differences between these parameters were tested for significance with the log‐rank test. All the factors found to be significant in univariate analysis were included in a multivariate analysis using a Cox proportional hazards model to determine independent prognostic factors for survival. All comparisons were two‐tailed, and a two‐tailed *P* value of ≤.05 was considered statistically significant. All statistical analyses were performed using the same software (SPSS Statistics version 22.0).

## 3 RESULTS

### 3.1 Patient characteristics

A total of 105 patients were enrolled in this study, and the female‐to‐male ratio was 2.5:1. The median age at diagnosis was 56 years (range, 18‐86 years). Overall, 86 patients (81%) had diseases involving the large salivary glands. The most commonly affected sites were the parotid gland (75/105, 71%), palate (13/105, 12%), and submandibular gland (10/105, 10%). Overall, 25 patients (24%) had involvement at multiple locations and 33 (31%) presented with nodal involvement. No cases of bone marrow involvement were identified.

In this study, 90% of the patients showed only local progressive swelling; only three patients (3%) had increased LDH, 23 had high levels of β2‐microglobulin (β2‐MG), and one had B symptoms. Overall, 52% of the patients had long‐term xerostomia, confirmed autoimmune diseases, or chronic parotitis. Common autoimmune diseases included SS (19/25, 76%), rheumatoid arthritis (4/25, 16%), and systemic lupus erythematosus (2/25, 8%). In addition, 69% of the pathological diagnoses were accompanied by a lymphoepithelial lesion. Only 17% of the patients underwent fine‐needle aspiration biopsy before the operation, whereas nearly half of them did not present evidence suggestive of malignancy.

**Figure 1:**
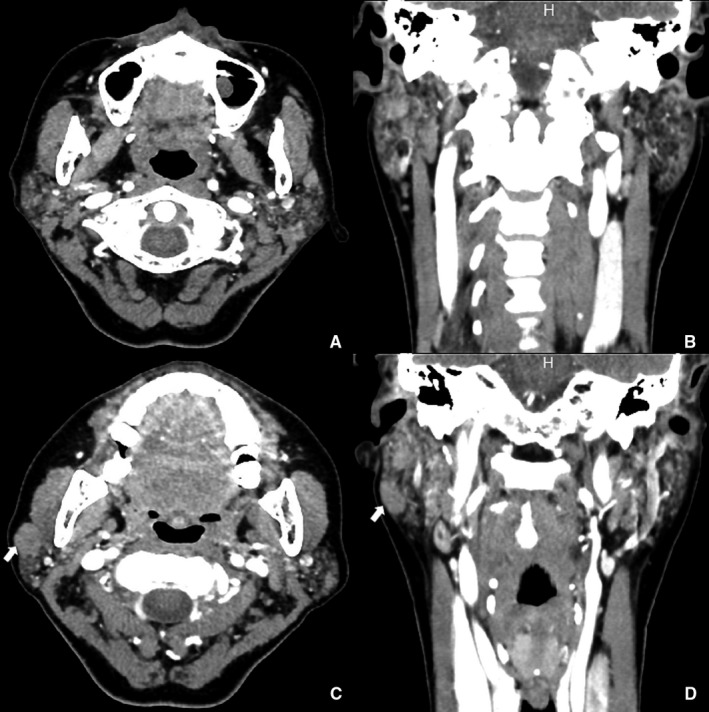
Enhanced computed tomography suggested MALT lymphoma in the right parotid gland (57‐year‐old female). Bilateral parotid glands were uneven with spotted high‐density focus, considered as a lymphoepithelial lesion or SS (A, B). Multiple solitary masses were present; the larger one (1.7 × 1.2 cm) was on the right (C, D)

**Figure 2:**
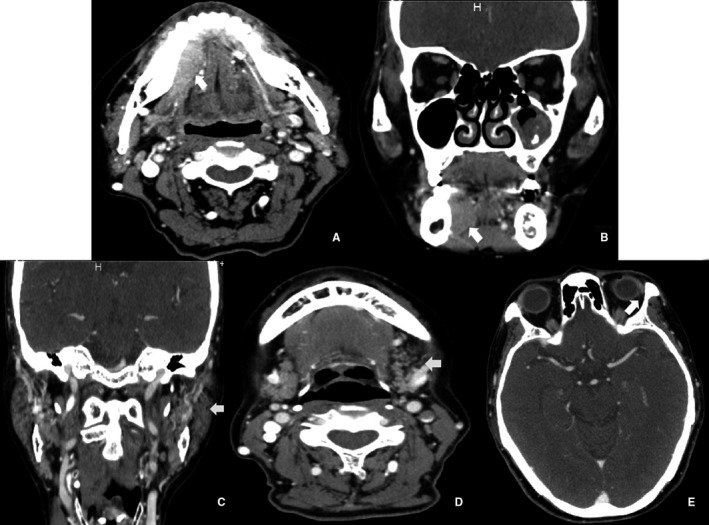
Enhanced computed tomography suggested MALT lymphoma in the right sublingual gland (70‐year‐old female). A spindle‐shaped soft‐tissue mass with an unclear boundary was observed in the right sublingual gland (A, B). Bilateral parotid glands and submandibular glands were uneven with spotted high‐density focus, considered as a lymphoepithelial lesion or SS. The left sublingual gland and bilateral lacrimal glands were atrophic (C–E)

In addition, preoperative enhanced computed tomography identified diffuse salivary gland lesions in 27% of the patients, potentially reflecting SS. Typical enhanced computed tomography of MALT lymphomas in the oromaxillofacial head and neck region is shown in Figures 1 and 2.

Among the 105 patients, two groups could be distinguished in terms of the dissemination of the disease at diagnosis: 70% of the patients had localized disease (stage I, 48 patients; stage II, 25 patients) and 30% had disseminated disease (stage III, 7 patients; stage IV, 25 patients). Patient characteristics and primary locations according to Ann Arbor staging are summarized in Table 1. The comparison between the two groups did not demonstrate any significant differences in any of the clinical characteristics (*P* > .05).

**Table 1:** Clinical characteristics and locations of 105 MALT lymphoma patients according to the stage of the disease

	All patients n (%)	Stage I‐II n (%)	Stage III‐IV n (%)	*P* value
Total	105 (100)	73 (70)	32 (30)	
Sex				.38
Male	30 (29)	19 (26)	11 (34)	
Female	75 (71)	54 (74)	21 (66)	
Age (y)				.17
<60	63 (60)	47 (64)	16 (50)	
≥60	42 (40)	26 (36)	16 (50)	
ECOG score				.98
0‐1	100 (95)	69 (95)	31 (97)	
2	5 (5)	4 (5)	1 (3)	
LDH				.22
Normal	102 (97)	72 (99)	30 (94)	
Elevated	3 (3)	1 (1)	2 (6)	
β*2‐MG*				.61
Normal	82 (78)	58 (79)	24 (75)	
Elevated	23 (22)	15 (21)	8 (25)	
B symptoms	1 (1)	—	1 (3)	.31
History				
Xerostomia	27 (26)	16 (22)	11 (34)	.18
Autoimmune diseases	25 (24)	15 (21)	10 (31)	.24
Parotitis	3 (3)	2 (3)	1 (3)	—
Histologic features				
Lymphoepithelial lesion	72 (69)	49 (67)	23 (72)	.63
MALT‐IPI				—
Low(0)	61 (58)	61 (84)	—	
Intermediate‐high(1‐2)	44 (42)	12 (16)	32 (30)	
Sites of primary tumors				—
One MALT‐organ localization	80 (76)	73 (100)	7 (22)	
Parotid	53 (50)	50 (68)	3 (9)	
Non‐parotid	27 (26)	23 (32)	4 (13)	
Nodal involvement	33 (31)	25 (34)	7 (22)	
Multiple MALT‐organ localizations	25 (24)	—	25 (78)	
Same organs	20 (19)	—	20 (63)	
Different organs	5 (5)	—	5 (15)	
Nodal involvement	1 (1)	—	1 (3)	

Abbreviations: β2‐MG, β2‐microglobulin; ECOG score, the Eastern Cooperative Oncology Group score; LDH, lactate dehydrogenase; MALT‐IPI, MALT Lymphoma International Prognostic Index.

### 3.2 Treatment

Patients were treated according to their disease stage and location; the treatments of patients are listed in Table 2. Local treatment included surgery (S) or local radiation therapy (RT), just be used for oromaxillofacial head and neck region; the average dose of RT was 20‐30 Gy. Systemic treatment included chemotherapy (CT) or rituximab (R). The chemotherapy combinations were cyclophosphamide, doxorubicin, vincristine, and prednisolone (CHOP) and cyclophosphamide, doxorubicin, vincristine, and prednisolone (COP).

**Table 2:** Treatment therapy according to the stage of 105 MALT lymphoma patients

	All patients n (%)	Stage I‐II n (%)	Stage III‐IV n (%)
Total	105 (100)	73 (100)	32 (100)
No active treatment	6 (6)	2 (3)	4 (13)
RT	5 (5)	3 (4)	2 (6)
RT+CT	4 (4)	—	4 (13)
RT+CT+R	3 (3)	1 (1)	2 (6)
Surgery	36 (34)	32 (44)	4 (13)
S+RT	23 (21)	20 (27)	3 (9)
S+CT	4 (4)	3 (4)	1 (3)
S+R	4 (4)	2 (3)	2 (6)
S+CT+R	5 (5)	4 (5)	1 (3)
S+RT+CT+R	6 (6)	5 (7)	1 (3)
Chemotherapy	4 (4)	1 (1)	3 (9)
Rituximab	1 (1)	—	1 (3)
CT+R	4 (4)	—	4 (13)

Abbreviations: CT, chemotherapy; R, rituximab; RT, local radiation therapy; S, surgery.

Overall, 75% (55/73) of the early patients received only local treatment; 24% (17/73) received systemic therapy after local treatment. In addition, 60% (19/32) of the advanced patients received systemic treatment after pathological diagnosis, whereas 28% (9/32) received only local treatment.

### 3.3 Response to treatment

Response to treatment was assessable in 97 patients (including 67 localized and 30 disseminated lymphoma patients). Overall, 78 patients had tumor‐free survival, five had survival with tumor, and 14 died. Deaths in seven (50%) were related to progression of disease or adverse reactions during the treatment among the 14 patients, whereas the others died of medical diseases.

CR after the first treatment was achieved in 78 patients (80%; 95% CI, 72%–89%). A significant difference was observed between the rate of CR after the first treatment in localized (58/67 patients, 87%) and disseminated (20/30 patients, 67%) lymphoma patients (χ^2^, *P* = .02). Patients who received rituximab alone and radiotherapy combined with chemotherapy had a CR rate of 100%; the CR rates of radiotherapy and chemotherapy were 77% and 40%, respectively. However, there was no significant difference among the rates of CR in the different treatment groups (*P* > .05).

In this study, 12 patients progressed with a median time of 4 years: seven patients progressed still in the oromaxillofacial head and neck region, four patients progressed to the lung (two patients), bone (one patient), or stomach (one patient), whereas one patient appeared to show systemic spread. Overall, four of the 12 patients died of disease progression, five achieved CR again after retreatment, and three were still under treatment. There was a significant difference between the rate of progression of localized (5/67 patients, 7%) and disseminated (7/30 patients, 23%) lymphoma patients (χ^2^, *P* = .04), but no significant difference was noted among the different treatment groups (*P* > .05).

### 3.4 Survival

**Figure 3:**
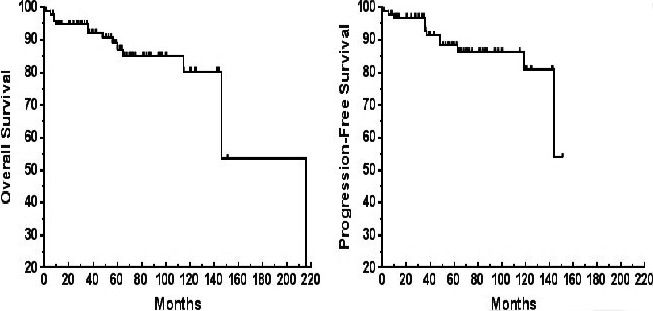
Survival curves for 97 patients with MALT lymphomas in the oromaxillofacial head and neck region

The median follow‐up was 5 years: 5 years for the localized lymphoma patient group and 4.3 years for the disseminated lymphoma patient group. The estimated 5‐year OS and PFS were 89% (95% CI, 82%–96%) and 88% (95% CI, 81%–96%), respectively (Figure 3).

**Figure 4:**
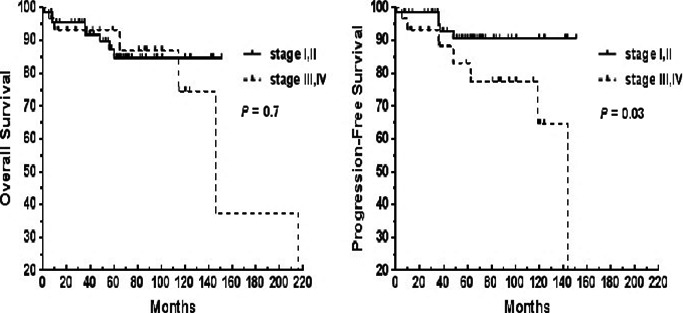
Survival curves for 67 patients with MALT lymphoma at Ann Arbor stages I/II and 30 patients with stages III/IV

As shown in Figure 4, the 5‐ and 10‐year OS rates of the localized lymphoma patients were both 85%, whereas those of the disseminated lymphoma patients were 93% and 75%, respectively. There was no significant difference in the OS of localized and disseminated patients with MALT lymphomas (*P* > .05). The 5‐ and 10‐year PFS of the localized lymphoma patients were both 91%, whereas those of the disseminated lymphoma patients were 83% and 65%, respectively; this difference between the two groups was significant (*P* = .03).

**Figure 5:**
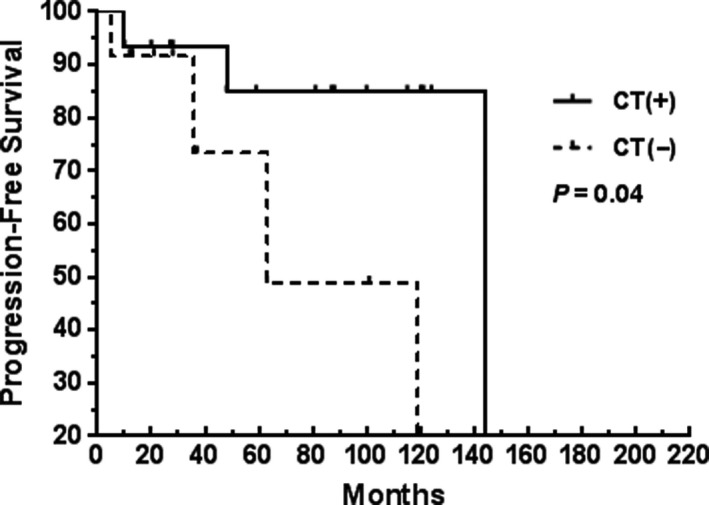
Impact of chemotherapy (CT) on the PFS of patients with MALT lymphoma at Ann Arbor stages III/IV: CT(+), 15 patients who received CT; CT(−), 15 patients who did not receive CT

**Figure 6:**
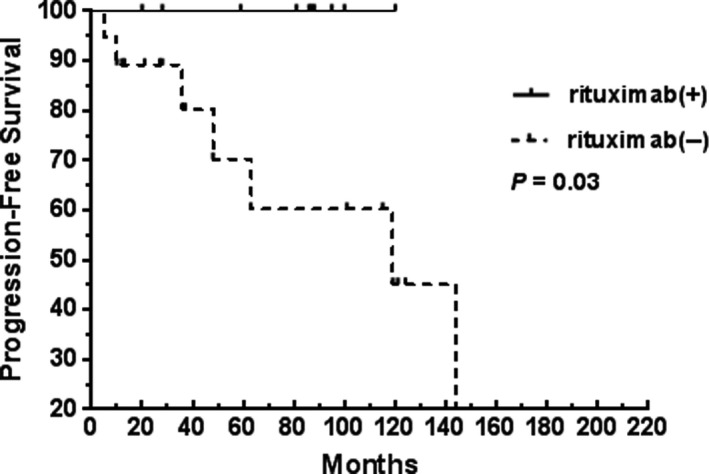
Impact of rituximab on the PFS of patients with MALT lymphoma at Ann Arbor stages III/IV: rituximab(+), 11 patients who received rituximab; rituximab(−), 19 patients who did not receive rituximab

In univariate analysis, Ann Arbor stages I/II, a single extranodal site of involvement, no residual primary lesion, chemotherapy, and low‐risk MALT‐IPI scores were the clinical features significantly associated with better OS or PFS (Table 3). Moreover, a better PFS was associated with the disseminated lymphoma patients treated with chemotherapy and rituximab. The 5‐year PFS rates of the chemotherapy and non‐chemotherapy groups in the disseminated lymphoma patients were 85% and 73%, respectively (Figure 5). Meanwhile, the 5‐year PFS rates of the rituximab and non‐rituximab groups in the disseminated lymphoma patients were 100% and 70%, respectively (Figure 6).

**Table 3:** Univariate analysis of prognostic factors for OS and PFS by univariate analysis of 97 MALT lymphoma patients

Prognostic factors	*P* value	
	OS	PFS
Ann Arbor stage III–IV	.695	.033
Increased serum LDH	.499	.504
Increased serum β2‐MG	.649	.648
Extranodal sites ≥2	.754	.037
Nodal disease	.081	.467
Residual primary lesion	.048	.218
Radiotherapy	.531	.845
Chemotherapy	.038	.799
Rituximab	.568	.056
MALT‐IPI ≥1	.013	.193

Abbreviations: β2‐MG, β2‐microglobulin; LDH, lactate dehydrogenase; MALT‐IPI, MALT Lymphoma International Prognostic Index; OS, overall survival; PFS, progression‐free survival**.**

In multivariate analysis, better OS was associated with a low‐risk MALT‐IPI score only, whereas better PFS was associated with Ann Arbor stages I/II only (Table 4).

**Table 4:** Multivariate analysis of prognostic factors for OS and PFS by Cox proportional hazards analysis of 97 MALT lymphoma patients

Prognostic factors	OS			PFS		
	Relative risk	95% CI	*P* value	Relative risk	95% CI	*P* value
MALT‐IPI ≥1	4.55	1.11‐18.69	.035			
Ann Arbor stage III–IV				3.25	1.03‐10.27	.045

Abbreviations: CI, confidence interval; MALT‐IPI, MALT Lymphoma International Prognostic Index; OS, overall survival; PFS, progression‐free survival**.**

## 4 DISCUSSION

Wenzel et al^19^ have reported 36 cases of MALT lymphoma in the head and neck region in patients with a median age of 54 years (range, 31‐82 years; a male‐to‐female ratio, 1:2); In another clinical analysis of 153 patients with NHLs in the head and neck region, the proportion of patients with MALT lymphoma was 9.2%, median age was 68 years (range, 32‐92 years), and male‐to‐female ratio was 1:1.64.^20^ Taken together, these findings indicate that MALT lymphomas in the oromaxillofacial head and neck region commonly occur in middle‐aged women; however, the reason for this female predominance remains unclear. We speculate that this may be influenced by the relatively higher rate of autoimmune diseases (especially SS) in women.^21^

In a previous multicenter clinical study on non‐gastric MALT lymphomas, the proportion of patients with advanced stage lymphomas and the involvement of multiple extranodal sites were close to the results of our study. In contrast, the majority of lymphomas were identified in the salivary glands, orbit, and thyroid gland; 3% of the patients had B symptoms, whereas 14% had bone marrow involvement.^7^ These results might be because our study was performed at a single center and can be influenced by sampling errors. In addition, it was proved that non‐gastric MALT lymphomas preferentially involve multiple extranodal sites compared with gastric MALT lymphoma, which might be associated with the higher affinity of lymphocytes to non‐gastric mucosa than extragastric MALT lymphomas,^22^ although this had no adverse effects on the overall prognosis.^23^

A clinical study in which 463 patients with SS were followed up over 25 years reported that 27 patients developed a distinct lymphoma, 24 showed involvement in the oromaxillofacial head and neck region, and six had MALT lymphoma.^24^ Overall, 24% of the patients with MALT lymphoma had confirmed autoimmune diseases in our study. It was confirmed that the damage of A20 protein encoded by TNFAIP3 leading to impaired control of NF‐κB activation in B cells continuously stimulated by autoimmunity enhanced the risk of lymphoma.^25^ In the analysis of autoimmune conditions and the risk of NHL and subtypes, SS and systemic lupus erythematosus were shown to be associated with a 30‐fold and eightfold increased risk of marginal zone lymphoma, respectively, and there was a 1000‐fold increase in the risk of parotid gland MALT lymphoma among SS patients. It was also suggested that such increased risks would not be decreased by the use of glucocorticoids and immunosuppressive agents.^26^ Our study also found that patients with MALT lymphoma with a history of SS had different control conditions of SS before the confirmation of lymphoma, suggesting that the treatment of autoimmune diseases does not reduce their risk of developing lymphoma.

Furthermore, patients with other types of malignant lymphomas in the oromaxillofacial head and neck region mostly report ulcers of skin and mucosa, necrosis of tissues, and loosening of teeth along with other malignant symptoms. It is also difficult to distinguish other benign or malignant tumors in the salivary glands and palate from MALT lymphomas. Therefore, for tumor patients with long‐term xerostomia, autoimmune diseases, or enhanced computed tomography suggestive of lymphoepithelial lesions or SS of the salivary glands, we should strongly consider the possibility of MALT lymphomas. It is necessary to initially identify benign and malignant tumors by fine‐needle aspiration biopsy^27^; however, the results of fine‐needle aspiration biopsy are not always completely reliable. Therefore, immunoglobulin clonal gene rearrangement^28^ or complete surgical resection is allowed in clinical practice in some cases.

The standard treatment for non‐gastric MALT lymphomas is not very clear. Surgery, radiotherapy, and chemotherapy alone as well as their combinations did not show significant differences in survival.^6^, ^7^, ^29^, ^30^, ^31^ The specific characteristics of MALT lymphomas in the oromaxillofacial head and neck region in terms of treatment and prognosis have also not been comprehensively reported in the recent clinical literature. In our study, more patients received local treatment than those in other studies,^29^, ^30^ which was related to many patients having involvement at resectable sites. Concerning the response to treatment, a higher complete remission rate and lower percentage of progression were noted in localized disease patients than in disseminated disease patients, potentially caused by a lower tumor burden. However, there was no significant difference among the treatment groups, although the complete remission rate of chemotherapy was the lowest, which might be related to the small size of each treatment group; this was consistent with the results of the study by Thieblemont.^6^ Other studies have showed that the CR rate of radiotherapy was 92%^32^ and radiotherapy could significantly reduce the rate of disease progression.^21^

The recurrence rate of MALT lymphoma in the head and neck region was high after local treatment (30%–43%) and systemic therapy, and long‐term monitoring appeared to be more appropriate in this context.^19^, ^33^, ^34^ Rituximab used to treat patients with MALT lymphoma with SS could not only control MALT lymphoma but also decrease the activity of SS.^35^ In addition, it was reported that rituximab and chemotherapy might have potential synergistic effects.^31^Our study found that systemic therapy could improve PFS in disseminated disease patients, confirming the benefit of systemic therapy for the prognosis of disseminated MALT lymphomas in the oromaxillofacial head and neck region.

According to the analysis of prognostic factors, we should consider more active treatment (such as chemotherapy and rituximab) to improve the prognosis of MALT lymphomas in the oromaxillofacial head and neck region with Ann Arbor stage III/IV or MALT‐IPI ≥1, although MALT lymphomas with multiple extranodal sites without bone marrow involvement might not be equivalent to disease dissemination.^36^ Furthermore, some studies have showed that β2‐microglobulin, anemia, bone marrow, and lymph node involvement were also factors affecting the prognosis of MALT lymphomas^5^, ^37^; however, no such effects on MALT lymphomas in the oromaxillofacial head and neck region were noted in our study; therefore, this requires further verification.

Chemotherapy regimens did not differ significantly in this study, which may be associated with the single chemotherapy regimen and the small number of patients at our center. Salar et al^38^ have found that bendamustine plus rituximab appeared to be an active and well‐tolerated first‐line treatment for patients with MALT lymphoma. The combination of rituximab and chlorambucil could also improve PFS in patients with MALT lymphoma.^39^ Serious side effects related to chemotherapy were reported in our study; therefore, it should be noted that side effects are unavoidable in chemotherapy in clinical practice. In recent years, basic research has made progress in shedding light on dysregulated pathways and immunological antitumor responses in indolent lymphoma.^40^ Substantial research related to nonchemotherapeutic agents, such as other anti‐CD20 monoclonal antibodies, anti‐CD22, or CD74 monoclonal antibodies,^41^, ^42^, ^43^ has achieved therapeutic advances. The development and application of these nonchemotherapeutic agents would make it possible to treat indolent lymphoma without chemotherapy.

## CONFLICT OF INTEREST

The authors have declared that no conflict of interest exist.

## AUTHOR CONTRIBUTIONS

Wei Guo and Guoxin Ren are corresponding author. Tian Zhang are first author and drafted the paper. Yunteng Wu and Houyu Ju were involved in statistical analysis. Guoxin Ren and Wei Guo modified the paper and designed this study concepts. All authors read and approved the final manuscript.

## Data Availability

The data that support the findings of this study are available from the corresponding author upon reasonable request.
